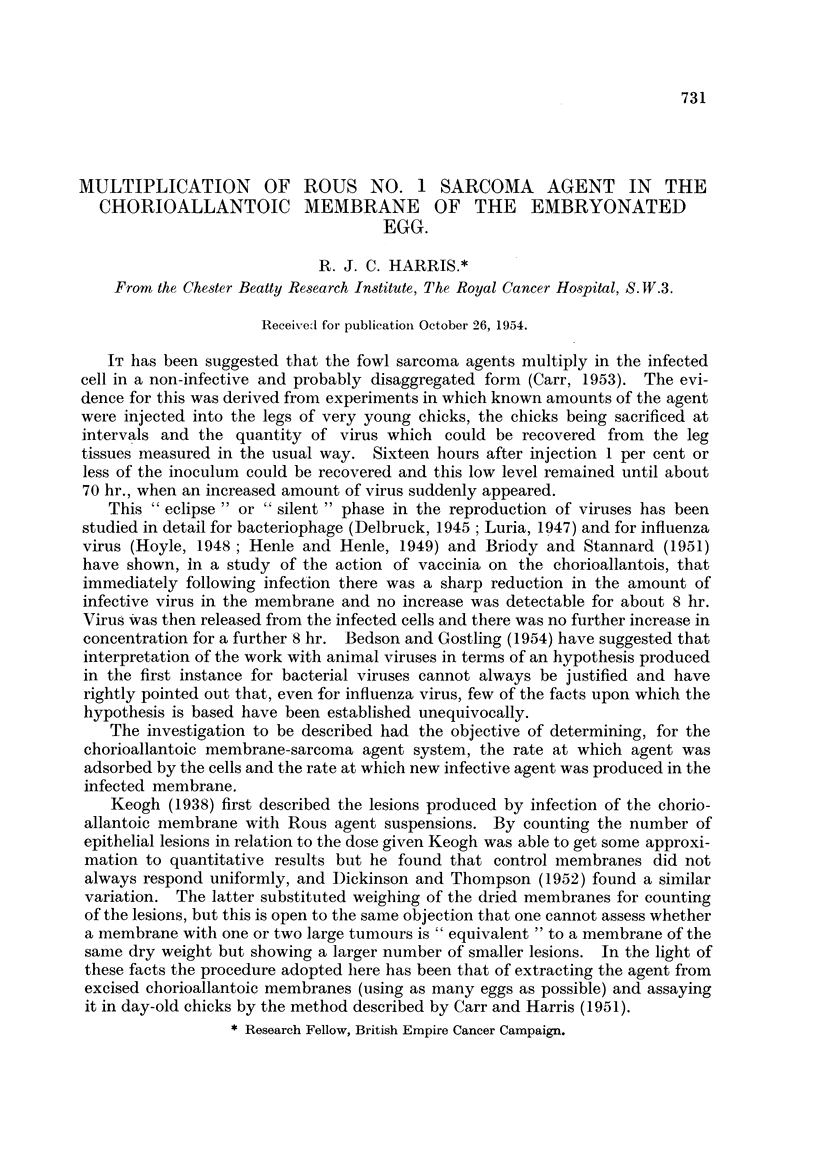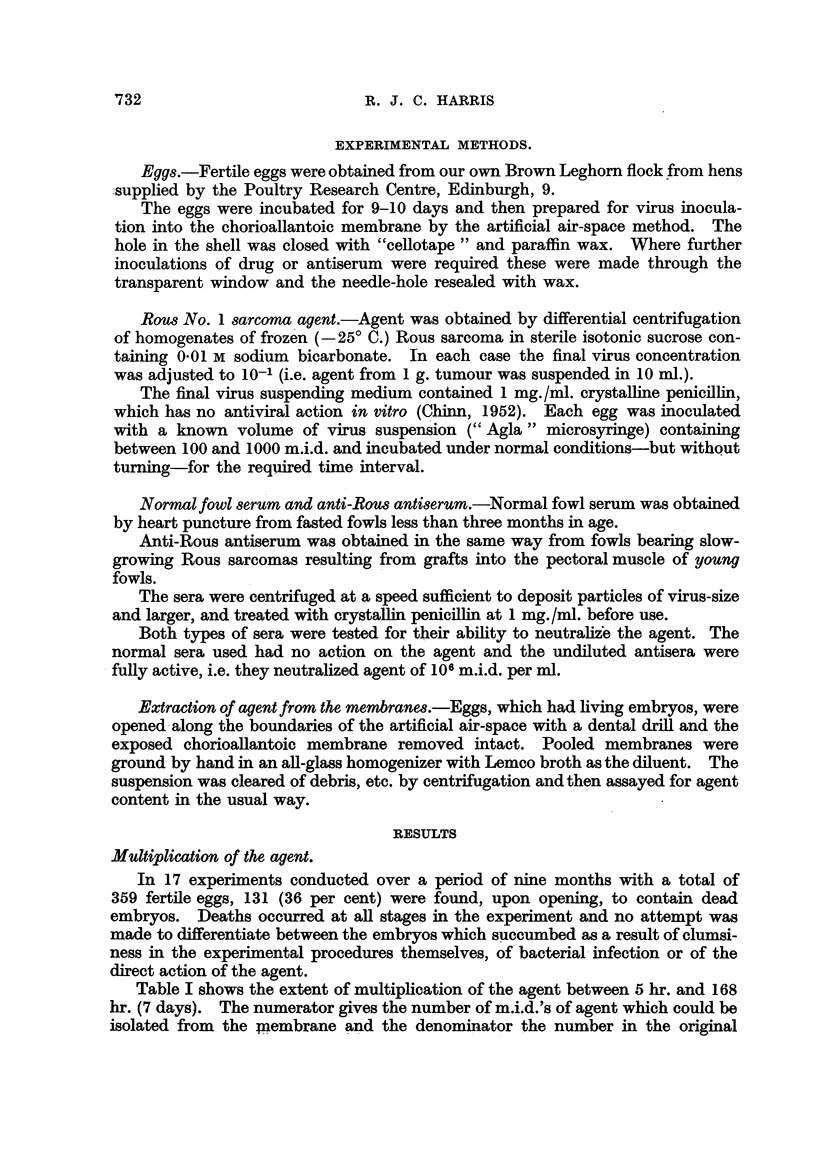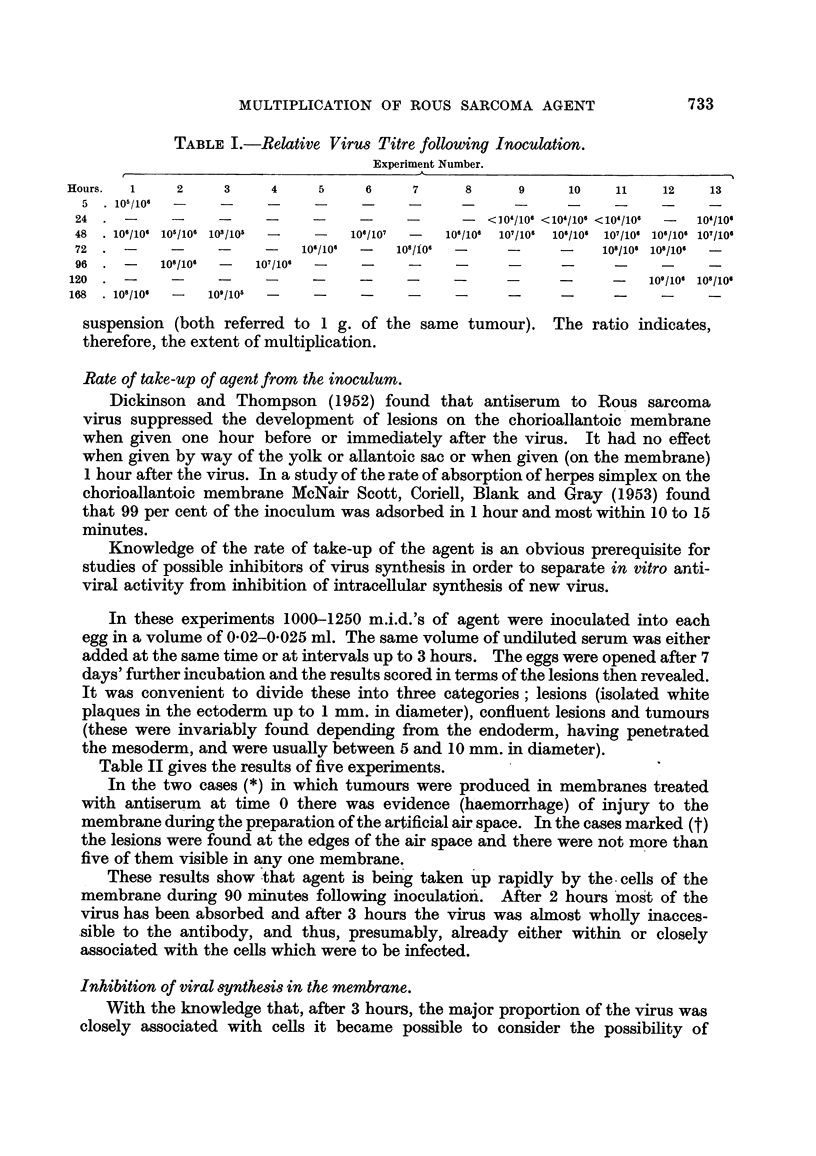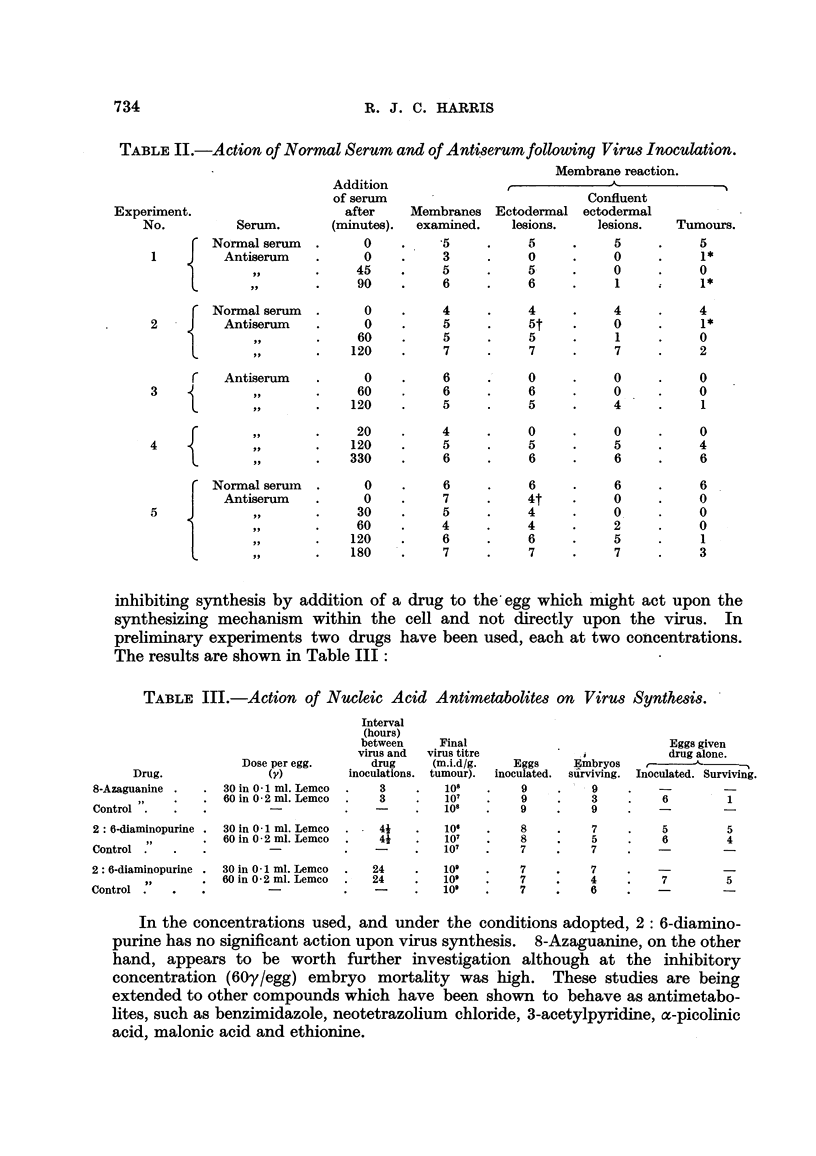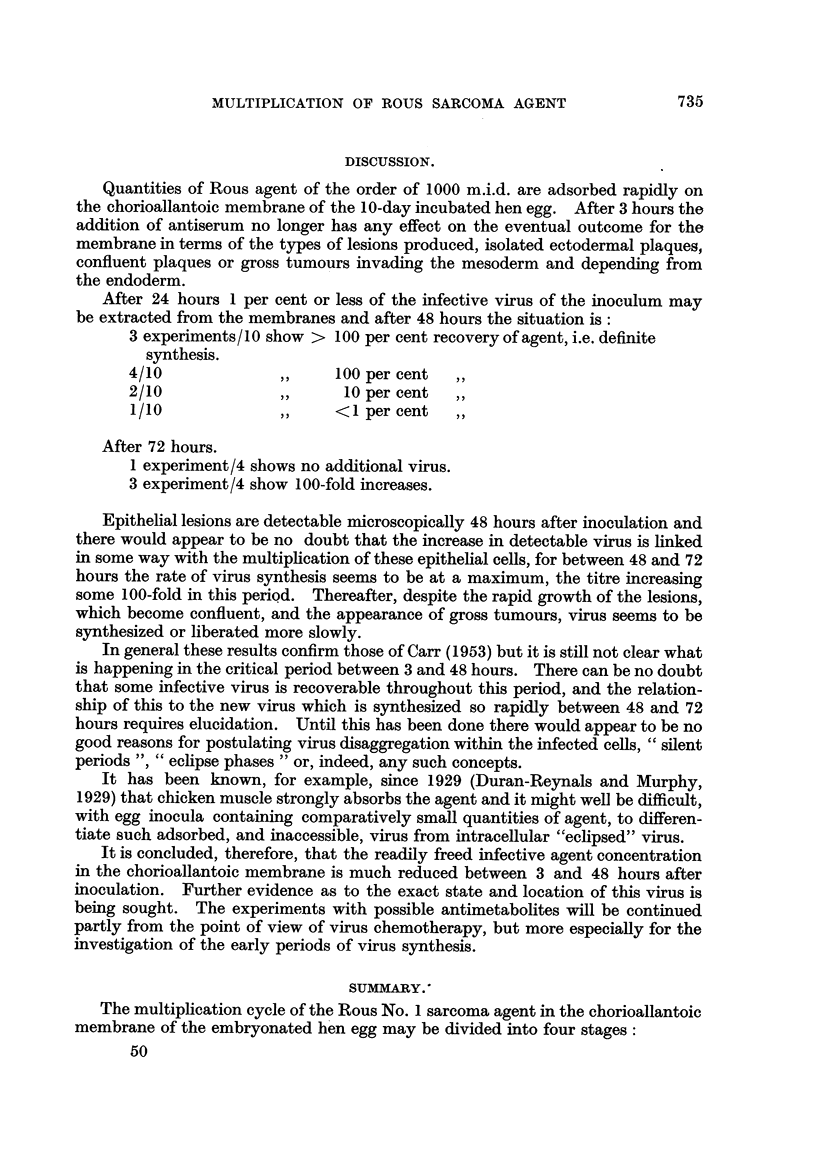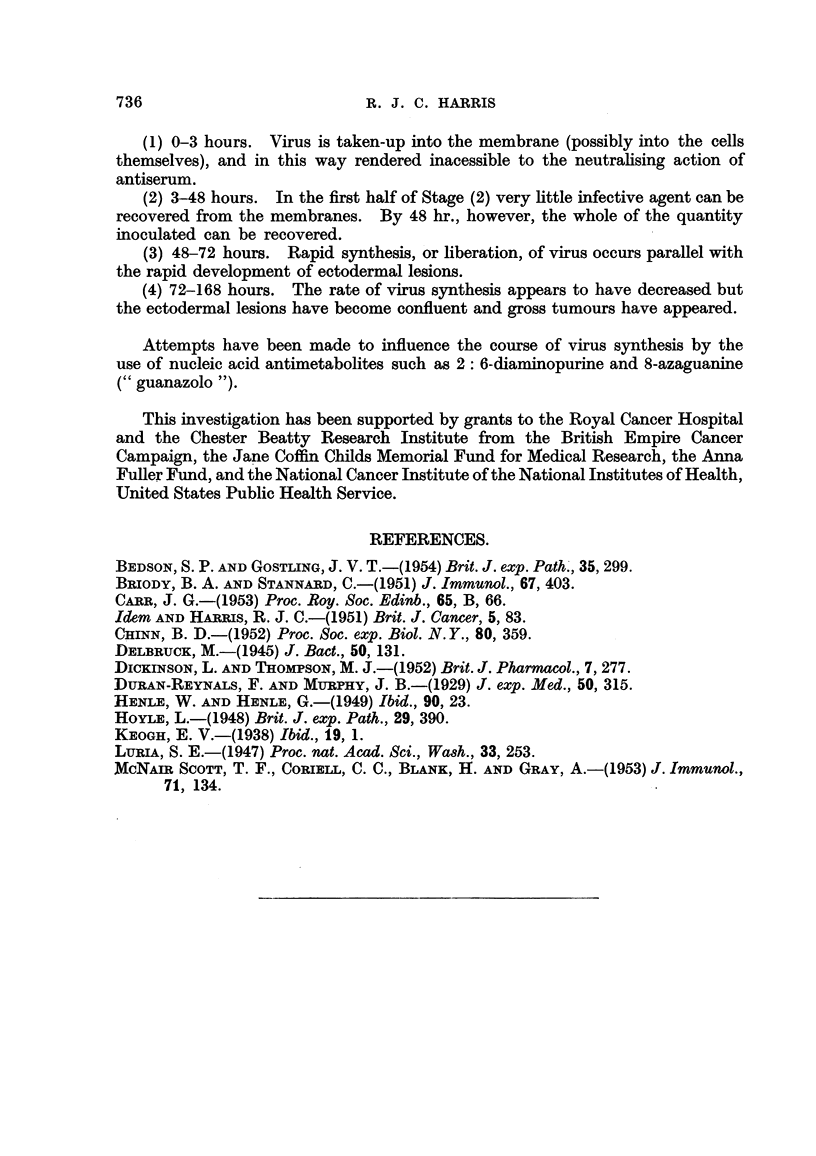# Multiplication of Rous No. 1 Sarcoma Agent in the Chorioallantoic Membrane of the Embryonated Egg

**DOI:** 10.1038/bjc.1954.81

**Published:** 1954-12

**Authors:** R. J. C. Harris


### MULTIPLICATION OF ROUS NO. 1 SARCOMA AGENT IN THE CHORIOALLANTOIC MEMBRANE OF THE EMBRYONATED EGG.

R. J. C. HARRIS.*

*From the Chester Beatty Research Institute, The Royal Cancer Hospital, S.W.3.*

Received for publication October 26, 1954.

It has been suggested that the fowl sarcoma agents multiply in the infected cell in a non-infective and probably disaggregated form (Carr, 1953). The evidence for this was derived from experiments in which known amounts of the agent were injected into the legs of very young chicks, the chicks being sacrificed at intervals and the quantity of virus which could be recovered from the leg tissues measured in the usual way. Sixteen hours after injection 1 per cent or less of the inoculum could be recovered and this low level remained until about 70 hr., when an increased amount of virus suddenly appeared.

This " eclipse " or " silent " phase in the reproduction of viruses has been studied in detail for bacteriophage (Delbruck, 1945 ; Luria, 1947) and for influenza virus (Hoyle, 1948 ; Henle and Henle, 1949) and Briody and Stannard (1951) have shown, in a study of the action of vaccinia on the chorioallantois, that immediately following infection there was a sharp reduction in the amount of infective virus in the membrane and no increase was detectable for about 8 hr. Virus was then released from the infected cells and there was no further increase in concentration for a further 8 hr. Bedson and Gostling (1954) have suggested that interpretation of the work with animal viruses in terms of an hypothesis produced in the first instance for bacterial viruses cannot always be justified and have rightly pointed out that, even for influenza virus, few of the facts upon which the hypothesis is based have been established unequivocally.

The investigation to be described had the objective of determining, for the chorioallantoic membrane-sarcoma agent system, the rate at which agent was adsorbed by the cells and the rate at which new infective agent was produced in the infected membrane.

Keogh (1938) first described the lesions produced by infection of the chorioallantoic membrane with Rous agent suspensions. By counting the number of epithelial lesions in relation to the dose given Keogh was able to get some approximation to quantitative results but he found that control membranes did not always respond uniformly, and Dickinson and Thompson (1952) found a similar variation. The latter substituted weighing of the dried membranes for counting of the lesions, but this is open to the same objection that one cannot assess whether a membrane with one or two large tumours is " equivalent " to a membrane of the same dry weight but showing a larger number of smaller lesions. In the light of these facts the procedure adopted here has been that of extracting the agent from excised chorioallantoic membranes (using as many eggs as possible) and assaying it in day-old chicks by the method described by Carr and Harris (1951).

* Research Fellow, British Empire Cancer Campaign.

### EXPERIMENTAL METHODS.

*Eggs.*—Fertile eggs were obtained from our own Brown Leghorn flock from hens supplied by the Poultry Research Centre, Edinburgh, 9.

The eggs were incubated for 9–10 days and then prepared for virus inoculation into the chorioallantoic membrane by the artificial air-space method. The hole in the shell was closed with "cellotape" and paraffin wax. Where further inoculations of drug or antiserum were required these were made through the transparent window and the needle-hole resealed with wax.

*Rous No. 1 sarcoma agent.*—Agent was obtained by differential centrifugation of homogenates of frozen (—25° C.) Rous sarcoma in sterile isotonic sucrose containing 0·01 M sodium bicarbonate. In each case the final virus concentration was adjusted to $10^{-1}$ (i.e. agent from 1 g. tumour was suspended in 10 ml.).

The final virus suspending medium contained 1 mg./ml. crystalline penicillin, which has no antiviral action *in vitro* (Chinn, 1952). Each egg was inoculated with a known volume of virus suspension ("Agla" microsyringe) containing between 100 and 1000 m.i.d. and incubated under normal conditions—but without turning—for the required time interval.

*Normal fowl serum and anti-Rous antiserum.*—Normal fowl serum was obtained by heart puncture from fasted fowls less than three months in age.

Anti-Rous antiserum was obtained in the same way from fowls bearing slow-growing Rous sarcomas resulting from grafts into the pectoral muscle of *young* fowls.

The sera were centrifuged at a speed sufficient to deposit particles of virus-size and larger, and treated with crystallin penicillin at 1 mg./ml. before use.

Both types of sera were tested for their ability to neutralize the agent. The normal sera used had no action on the agent and the undiluted antisera were fully active, i.e. they neutralized agent of $10^6$ m.i.d. per ml.

*Extraction of agent from the membranes.*—Eggs, which had living embryos, were opened along the boundaries of the artificial air-space with a dental drill and the exposed chorioallantoic membrane removed intact. Pooled membranes were ground by hand in an all-glass homogenizer with Lemco broth as the diluent. The suspension was cleared of debris, etc. by centrifugation and then assayed for agent content in the usual way.

### RESULTS

### *Multiplication of the agent.*

In 17 experiments conducted over a period of nine months with a total of 359 fertile eggs, 131 (36 per cent) were found, upon opening, to contain dead embryos. Deaths occurred at all stages in the experiment and no attempt was made to differentiate between the embryos which succumbed as a result of clumsiness in the experimental procedures themselves, of bacterial infection or of the direct action of the agent.

Table I shows the extent of multiplication of the agent between 5 hr. and 168 hr. (7 days). The numerator gives the number of m.i.d.'s of agent which could be isolated from the membrane and the denominator the number in the original

TABLE I.—*Relative Virus Titre following Inoculation.*

| Hours. | Experiment Number. 1 | 2 | 3 | 4 | 5 | 6 | 7 | 8 | 9 | 10 | 11 | 12 | 13 |
|---|---|---|---|---|---|---|---|---|---|---|---|---|---|
| 5 | $10^5/10^6$ | — | — | — | — | — | — | — | — | — | — | — | — |
| 24 | — | — | — | — | — | — | — | — | $<10^4/10^6$ | $<10^4/10^6$ | $<10^4/10^6$ | — | $10^4/10^6$ |
| 48 | $10^6/10^6$ | $10^5/10^6$ | $10^3/10^5$ | — | — | $10^6/10^7$ | — | $10^6/10^6$ | $10^7/10^6$ | $10^6/10^6$ | $10^7/10^6$ | $10^6/10^6$ | $10^7/10^6$ |
| 72 | — | — | — | — | $10^6/10^6$ | — | $10^8/10^6$ | — | — | — | $10^8/10^6$ | $10^8/10^6$ | — |
| 96 | — | $10^8/10^6$ | — | $10^7/10^6$ | — | — | — | — | — | — | — | — | — |
| 120 | — | — | — | — | — | — | — | — | — | — | — | $10^9/10^6$ | $10^8/10^6$ |
| 168 | $10^8/10^6$ | — | $10^9/10^5$ | — | — | — | — | — | — | — | — | — | — |

suspension (both referred to 1 g. of the same tumour). The ratio indicates, therefore, the extent of multiplication.

*Rate of take-up of agent from the inoculum.*

Dickinson and Thompson (1952) found that antiserum to Rous sarcoma virus suppressed the development of lesions on the chorioallantoic membrane when given one hour before or immediately after the virus. It had no effect when given by way of the yolk or allantoic sac or when given (on the membrane) 1 hour after the virus. In a study of the rate of absorption of herpes simplex on the chorioallantoic membrane McNair Scott, Coriell, Blank and Gray (1953) found that 99 per cent of the inoculum was adsorbed in 1 hour and most within 10 to 15 minutes.

Knowledge of the rate of take-up of the agent is an obvious prerequisite for studies of possible inhibitors of virus synthesis in order to separate *in vitro* antiviral activity from inhibition of intracellular synthesis of new virus.

In these experiments 1000–1250 m.i.d.'s of agent were inoculated into each egg in a volume of 0·02–0·025 ml. The same volume of undiluted serum was either added at the same time or at intervals up to 3 hours. The eggs were opened after 7 days' further incubation and the results scored in terms of the lesions then revealed. It was convenient to divide these into three categories ; lesions (isolated white plaques in the ectoderm up to 1 mm. in diameter), confluent lesions and tumours (these were invariably found depending from the endoderm, having penetrated the mesoderm, and were usually between 5 and 10 mm. in diameter).

Table II gives the results of five experiments.

In the two cases (*) in which tumours were produced in membranes treated with antiserum at time 0 there was evidence (haemorrhage) of injury to the membrane during the preparation of the artificial air space. In the cases marked (†) the lesions were found at the edges of the air space and there were not more than five of them visible in any one membrane.

These results show that agent is being taken up rapidly by the cells of the membrane during 90 minutes following inoculation. After 2 hours most of the virus has been absorbed and after 3 hours the virus was almost wholly inaccessible to the antibody, and thus, presumably, already either within or closely associated with the cells which were to be infected.

*Inhibition of viral synthesis in the membrane.*

With the knowledge that, after 3 hours, the major proportion of the virus was closely associated with cells it became possible to consider the possibility of

TABLE II.—*Action of Normal Serum and of Antiserum following Virus Inoculation.*

| Experiment. No. | Serum. | Addition of serum after (minutes). | Membranes examined. | Membrane reaction. Ectodermal lesions. | Membrane reaction. Confluent ectodermal lesions. | Membrane reaction. Tumours. |
|---|---|---|---|---|---|---|
| 1 | Normal serum | 0 | 5 | 5 | 5 | 5 |
| | Antiserum | 0 | 3 | 0 | 0 | 1* |
| | ,, | 45 | 5 | 5 | 0 | 0 |
| | ,, | 90 | 6 | 6 | 1 | 1* |
| 2 | Normal serum | 0 | 4 | 4 | 4 | 4 |
| | Antiserum | 0 | 5 | 5† | 0 | 1* |
| | ,, | 60 | 5 | 5 | 1 | 0 |
| | ,, | 120 | 7 | 7 | 7 | 2 |
| 3 | Antiserum | 0 | 6 | 0 | 0 | 0 |
| | ,, | 60 | 6 | 6 | 0 | 0 |
| | ,, | 120 | 5 | 5 | 4 | 1 |
| 4 | ,, | 20 | 4 | 0 | 0 | 0 |
| | ,, | 120 | 5 | 5 | 5 | 4 |
| | ,, | 330 | 6 | 6 | 6 | 6 |
| 5 | Normal serum | 0 | 6 | 6 | 6 | 6 |
| | Antiserum | 0 | 7 | 4† | 0 | 0 |
| | ,, | 30 | 5 | 4 | 0 | 0 |
| | ,, | 60 | 4 | 4 | 2 | 0 |
| | ,, | 120 | 6 | 6 | 5 | 1 |
| | ,, | 180 | 7 | 7 | 7 | 3 |

inhibiting synthesis by addition of a drug to the egg which might act upon the synthesizing mechanism within the cell and not directly upon the virus. In preliminary experiments two drugs have been used, each at two concentrations. The results are shown in Table III :

TABLE III.—*Action of Nucleic Acid Antimetabolites on Virus Synthesis.*

| Drug. | Dose per egg. ($\gamma$) | Interval (hours) between virus and drug inoculations. | Final virus titre (m.i.d/g. tumour). | Eggs inoculated. | Embryos surviving. | Eggs given drug alone. Inoculated. | Eggs given drug alone. Surviving. |
|---|---|---|---|---|---|---|---|
| 8-Azaguanine | 30 in 0·1 ml. Lemco | 3 | $10^8$ | 9 | 9 | — | — |
| ,, | 60 in 0·2 ml. Lemco | 3 | $10^7$ | 9 | 3 | 6 | 1 |
| Control | — | — | $10^8$ | 9 | 9 | — | — |
| 2 : 6-diaminopurine | 30 in 0·1 ml. Lemco | 4½ | $10^6$ | 8 | 7 | 5 | 5 |
| ,, | 60 in 0·2 ml. Lemco | 4½ | $10^7$ | 8 | 5 | 6 | 4 |
| Control | — | — | $10^7$ | 7 | 7 | — | — |
| 2 : 6-diaminopurine | 30 in 0·1 ml. Lemco | 24 | $10^9$ | 7 | 7 | — | — |
| ,, | 60 in 0·2 ml. Lemco | 24 | $10^9$ | 7 | 4 | 7 | 5 |
| Control | — | — | $10^9$ | 7 | 6 | — | — |

In the concentrations used, and under the conditions adopted, 2 : 6-diaminopurine has no significant action upon virus synthesis. 8-Azaguanine, on the other hand, appears to be worth further investigation although at the inhibitory concentration ($60\gamma$/egg) embryo mortality was high. These studies are being extended to other compounds which have been shown to behave as antimetabolites, such as benzimidazole, neotetrazolium chloride, 3-acetylpyridine, $\alpha$-picolinic acid, malonic acid and ethionine.

### DISCUSSION.

Quantities of Rous agent of the order of 1000 m.i.d. are adsorbed rapidly on the chorioallantoic membrane of the 10-day incubated hen egg. After 3 hours the addition of antiserum no longer has any effect on the eventual outcome for the membrane in terms of the types of lesions produced, isolated ectodermal plaques, confluent plaques or gross tumours invading the mesoderm and depending from the endoderm.

After 24 hours 1 per cent or less of the infective virus of the inoculum may be extracted from the membranes and after 48 hours the situation is :

3 experiments/10 show $>$ 100 per cent recovery of agent, i.e. definite synthesis.
4/10 ,, 100 per cent ,,
2/10 ,, 10 per cent ,,
1/10 ,, $<1$ per cent ,,

After 72 hours.
1 experiment/4 shows no additional virus.
3 experiment/4 show 100-fold increases.

Epithelial lesions are detectable microscopically 48 hours after inoculation and there would appear to be no doubt that the increase in detectable virus is linked in some way with the multiplication of these epithelial cells, for between 48 and 72 hours the rate of virus synthesis seems to be at a maximum, the titre increasing some 100-fold in this period. Thereafter, despite the rapid growth of the lesions, which become confluent, and the appearance of gross tumours, virus seems to be synthesized or liberated more slowly.

In general these results confirm those of Carr (1953) but it is still not clear what is happening in the critical period between 3 and 48 hours. There can be no doubt that some infective virus is recoverable throughout this period, and the relationship of this to the new virus which is synthesized so rapidly between 48 and 72 hours requires elucidation. Until this has been done there would appear to be no good reasons for postulating virus disaggregation within the infected cells, " silent periods ", " eclipse phases " or, indeed, any such concepts.

It has been known, for example, since 1929 (Duran-Reynals and Murphy, 1929) that chicken muscle strongly absorbs the agent and it might well be difficult, with egg inocula containing comparatively small quantities of agent, to differentiate such adsorbed, and inaccessible, virus from intracellular "eclipsed" virus.

It is concluded, therefore, that the readily freed infective agent concentration in the chorioallantoic membrane is much reduced between 3 and 48 hours after inoculation. Further evidence as to the exact state and location of this virus is being sought. The experiments with possible antimetabolites will be continued partly from the point of view of virus chemotherapy, but more especially for the investigation of the early periods of virus synthesis.

### SUMMARY.

The multiplication cycle of the Rous No. 1 sarcoma agent in the chorioallantoic membrane of the embryonated hen egg may be divided into four stages :

(1) 0–3 hours. Virus is taken-up into the membrane (possibly into the cells themselves), and in this way rendered inaccessible to the neutralising action of antiserum.

(2) 3–48 hours. In the first half of Stage (2) very little infective agent can be recovered from the membranes. By 48 hr., however, the whole of the quantity inoculated can be recovered.

(3) 48–72 hours. Rapid synthesis, or liberation, of virus occurs parallel with the rapid development of ectodermal lesions.

(4) 72–168 hours. The rate of virus synthesis appears to have decreased but the ectodermal lesions have become confluent and gross tumours have appeared.

Attempts have been made to influence the course of virus synthesis by the use of nucleic acid antimetabolites such as 2 : 6-diaminopurine and 8-azaguanine (" guanazolo ").

This investigation has been supported by grants to the Royal Cancer Hospital and the Chester Beatty Research Institute from the British Empire Cancer Campaign, the Jane Coffin Childs Memorial Fund for Medical Research, the Anna Fuller Fund, and the National Cancer Institute of the National Institutes of Health, United States Public Health Service.

### REFERENCES.

Bedson, S. P. and Gostling, J. V. T.—(1954) *Brit. J. exp. Path.*, **35**, 299.
Briody, B. A. and Stannard, C.—(1951) *J. Immunol.*, **67**, 403.
Carr, J. G.—(1953) *Proc. Roy. Soc. Edinb.*, **65**, B, 66.
*Idem* and Harris, R. J. C.—(1951) *Brit. J. Cancer*, **5**, 83.
Chinn, B. D.—(1952) *Proc. Soc. exp. Biol. N.Y.*, **80**, 359.
Delbruck, M.—(1945) *J. Bact.*, **50**, 131.
Dickinson, L. and Thompson, M. J.—(1952) *Brit. J. Pharmacol.*, **7**, 277.
Duran-Reynals, F. and Murphy, J. B.—(1929) *J. exp. Med.*, **50**, 315.
Henle, W. and Henle, G.—(1949) *Ibid.*, **90**, 23.
Hoyle, L.—(1948) *Brit. J. exp. Path.*, **29**, 390.
Keogh, E. V.—(1938) *Ibid.*, **19**, 1.
Luria, S. E.—(1947) *Proc. nat. Acad. Sci., Wash.*, **33**, 253.
McNair Scott, T. F., Coriell, C. C., Blank, H. and Gray, A.—(1953) *J. Immunol.*, **71**, 134.